# The conversion of forestland into agricultural land without appropriate measures to conserve SOM leads to the degradation of physical and rheological soil properties

**DOI:** 10.1038/s41598-020-70464-6

**Published:** 2020-08-12

**Authors:** Miodrag Tolimir, Branka Kresović, Ljubomir Životić, Snežana Dragović, Ranko Dragović, Zorica Sredojević, Boško Gajić

**Affiliations:** 1 Maize Research Institute, “Zemun Polje’’, Slobodana Bajića 1, 11185 Belgrade, Serbia; 2 Faculty of Agriculture, University of Belgrade, Nemanjina 6, 11080 Belgrade, Serbia; 3 Department of Chemical Dynamics and Permanent Education, „VINČA“ Institute of Nuclear Sciences - National Institute of thе Republic of Serbia, University of Belgrade, Mike Petrovića Alasa 12-14, 11351 Vinča, Belgrade, Serbia; 4 Faculty of Sciences and Mathematics, Department of Geography, University of Niš, Višegradska 33, 18000 Niš, Serbia

**Keywords:** Agroecology, Environmental impact

## Abstract

This study was conducted to compare soil particle density (ρ_s_), soil total porosity (TP), liquid limit (LL), plastic limit (PL), and plasticity index, and their relations with soil organic matter (SOM), of non-carbonate silty clay Fluvisols under different land uses. Three neighboring land uses were studied: native deciduous forest, arable land, and meadow, managed in the same way for more than 100 years. Soil was collected from 27 soil profiles and from three depths (0–15, 15–30 and 30–45 cm). Land use caused statistically significant but different impacts on soil properties, particularly in the topsoil. The forest topsoil measured the lowest ρ_s_ and bulk density (ρ_b_) but the highest SOM and soil water content at PL, compared to meadow and arable soil. Statistically significant linear relationship was observed with the SOM content and ρ_s_ (− 0.851**), ρ_b_ (− 0.567**), calculated TP (0.567**) and measured TP (− 0.280**). There was a nonlinear relationship between SOM and LL (0.704**) and PL (0.845**) at the topsoil. The findings suggested that SOM content strongly affected ρ_s_, ρ_b_, TP, LL and LP. This regional study showed that the conversion of forestland into agricultural land without appropriate measures to conserve SOM leads to the degradation of physical and rheological soil properties.

## Introduction

There is concern on a global scale that current soil management practices have an adverse effect on soils and that for some soils and land use combinations current practices might not be sustainable^1^. Serbia experienced considerable land use changes in the first half of the nineteenth century due to an increased demand for agricultural products. This led to extensive cutting of native deciduous forests and conversion into arable land and meadows^2^. Deforestation is a visible land degradation driver in the environment. It causes multiple changes that can manifest through the reduction of soil chemical and physical properties, leading to soil quality decline and continuining reduction of productivity. The transformation of soil organic matter (SOM) in cultivated soils, if no organic fertilizers are applied, leads to soil degradation and eventual inability to ensure sustainable agricultural production. Pérez-Bejarano^3^ found that organic carbon content was well related with most physical, chemical and biochemical soil properties. Physical soil properties, such as bulk density (ρ_b_), particle density (ρ_s_) and soil total porosity (TP), have a strong impact on the exchange of energy and matter between the atmosphere and the pedosphere^4^. Particle density data are of major importance in soil research because ρ_s_ is needed to estimate TP, rate of particle sedimentation, relative saturation, thermal conductivity, heat capacity, and volumetric water-to-air ratios. Specific uses of ρ_s_ include modeling of water, air and heat flow processes, as well as transport of chemicals in the soil^5,6^, or are used to calibrate soil moisture sensors^7^. However, ρ_s_ is usually not determined experimentally, but assumed to vary from 2.60–2.70 Mg m^–3^ or to be equal to a constant value of 2.65 Mg m^–3^^8^. This ρ_s_ value is acceptable for some soils, but variations in the composition of soil solids, such as a reduced SOM content, can lead to a substantial increase in ρ_s_ because the density of SOM is lower than that of mineral particles. Consequently, the determination of soil properties, which requires ρ_s_ as an input, is generally based on an assumed constant value of ρ_s_.

Different land uses are not expected to have a significant effect on ρ_s_, but changes in SOM due to soil management types can alter ρ_s_. For example, the conversion of forests into meadows and arable land reduces soil organic carbon pools^1,9^. The effect of this reduced SOM on ρ_s_ has not been well documented in the literature but the general assumption is that any change in ρ_s_ would be negligible^6^, which might not be exact. Only a few studies that compare the values of ρ_s_ as a result of SOM changes due to different long-term (> 100 years) land uses have been reported worldwide.

Total soil porosity is one of the most important properties determined directly by means of ρ_s_ which is often calculated using assumed standard value of ρ_s_^9,10^. Thus, the potential effect of land use on ρ_s_ was not taken into account. Characterization of ρ_s_ for different soil management practices, with variable SOM of the same soil, is a priority area of research because ρ_s_ variations due to different land uses, in cases where soil properties are assessed based on ρ_s_, are seldom quantified in the literature^6^.

SOM variations due to long-term land use and tillage impacts also affect the soil plastic limits, because of the high water absorption capacity of organic matter and interactions with soil mineral particles, which affect the bond strength and surface tension properties of soils^11^. However, plastic limits are rarely part of routine soil analyses^12^. Plastic limits can be used to determine the optimal water content for tillage without adversely affecting the soil structure^13^ and are also useful in assessing the impact of long-term land use and tillage on the mechanical and rheological behavior of soil. The correlation between SOM and plastic limits can be strong, weak or non-existent, depending on other soil properties^14–17^, such as origin of SOM, clay type and mineralogy, soil texture, nature of exchanged cations, and soil-crop management. These conflicting conclusions necessitate additional research, to shed more light on the correlation between SOM and plastic limits^6,17^. Quantitative LL and PL data are needed in areas where such information is not available^18^. The negative effects of long-term tillage on soil physical properties, and resulting soil degradation have not been widely recognized in the world and thus are poorly documented.

Consequently, the present research focuses on quantification of the effects of long-term land use changes and agricultural practices on soil degradation for three land uses including native deciduous forest and deforested area used as meadow and arable land through: (1) comparison of ρ_s_ and soil consistency of non-carbonate, silty clay Fluvisols; (2) comparison of TP determined using constant value of ρ_s_ and measured values of ρ_s_; and (3) evaluating the relationships among SOM, ρ_s_ and plastic limits. We hypothesized that land use is dominant factor controlling SOM and that SOM content strongly affects various soil physical properties (ρ_s_, ρ_b_ and TP) and plastic limits of non-calcareous Fluvisol of western Serbia. The information gathered from this research will improve knowledge about how ρ_s_, TP and consistency of soil vary as a function of land use. The results enable a better understanding of deforestation as a driver of human-induced soil degradation and necessity to utilize sustainable land management practices.

## Materials and methods

### Study area and land use systems

The research reported in this paper was conducted in the Kolubara River valley, Serbia (44° 30′ 43″ N, 20° 14′ 52″ E, elevation 87 m), in mid-June 2016. Three locations in the central part of the Kolubara River Basin were selected to determine how the soil responded to different land uses after more than 100 years. Each location included a native deciduous forest, permanent meadow and arable land of same parent material, topography and soil textural class. The distance among forest, meadow and arable land on each location was 50–100 m. All the plots were in a natural environment and land use was the only difference that might have affected soil properties.

The native deciduous forests were largely oak and ash communities (*As. Querceto-Fraxinetum serbicum* Rud.). In the natural meadows, the dominant species were orchardgrass (*Dactylis glomerata* L.), black medick (*Medicago lupulina*) and sweet pea (*Lathyrus sp*.), which have been used only for hay in the past 100 years. Usually were two mowing events yearly. A compound fertilizer of 15–15–15 in granular form was applied to the meadows at a rate of 22 kg pure N, 22 kg P_2_O_5_, and 22 kg K_2_O ha^–1^ at the start of vegetation. The arable land was moldboard plowed to a depth of 20–25 cm, followed by disking and harrowing, which are typical agricultural practices in the region. Cropping systems mainly include winter wheat (*Triticum aestivum* L.) and corn (*Zea mays* L.) annual rotation. Crop residues are collected and used as animal feed. The fertilizer doses for winter wheat, and corn were around N_91_ P_37_ K_37_, and N_120_ P_52_ K_52_ kg ha^−1^, respectively, based on higher yield goal. The fertilizer requirements were calculated on soil test basis. Manure is added every 3–4 years in very small doses. Pest, disease and weed management followed common practices. Farmers stated that land use in the study area have not changed for more than 100 years. This made it possible to assess the impact of land uses, especially meadows and arable soils, on SOM, ρs, TP and consistency of the soil, compared to forest over a period of around 100 years. Each location was tested by a hand auger, to determine whether the soils were homogeneous in terms of soil type, profile development, and texture of surface and subsurface soil layers.

The soil at all three locations was non-carbonate silty clay Fluvisols^19^. Average content of sand (2.00–0.05 mm), silt (0.05–0.002 mm) and clay (< 0.002 mm) were 5–11%, 48–56%, and 41–48%, respectively. The Fluvisols were formed over slightly carbonate, low-humus (< 0.5%) loamy alluvial sediments of the Kolubara River and are suitable for farming. This particular soil type is among the most fertile in Serbia and large parts of South East Europe. It occupies a land area of > 500,000 ha (13% of all arable land in Serbia, of which 10,000 ha is in the Kolubara valley). The multiyear mean annual precipitation in the study area is 730 mm and the multiyear average temperature 11 °C.

### Soil sampling and analyses

Three soil profile pits for each land use were dug at each of the three locations for soil sampling. They were spaced 10–15 m apart. Therefore, there were nine soil profile pits from each land use system (3 locations × 3 pits = 9 pits/land use system). Approximately 1,500 g of disturbed samples were collected from the same depths to determine SOM, ρ_s_, PL and LL. Disturbed soil samples were air-dried and passed through a 2 mm sieve for laboratory analysis. Undisturbed soil samples (diameter 5.4 cm and length 4.4 cm) for ρ_b_ analyses were collected by means of steel cylindres, five each from different depths: 0–15, 15–30 and 30–45 cm. The ρ_b_ was determined from cores dried at 105 °C to constant mass as the ratio of dry soil sample and volume of the core (100 cm^3^)^20^. The ρ_s_ was determined as the ratio of dry weight of the soil to the volume of soil particles using the standard water pycnometer method without destroying SOM^20^. The Casagrande method was used to determine LL^21^. Approximately 200–300 g of air-dried soil sample was wetted with distilled water remolded and put in the brass cup of Casagrande apparatus. The sample was divided in two by standard grooving tool. The cup was raised 1 cm above the hard rubber base by a crank and dropped freely at the rate of two drops per second until the two parts of the soil came into contact at the bottom of the groove over a length of 1.3 cm. The number of blows required to do this was recorded. After that, more distilled water was added to the soil sample and the procedure was repeated for other two water contents to give a range of blows lying between 50 and 10. The procedure followed was to plot the moisture content versus the logarithm of the blow content for each trial on semilog graph paper. The gravimetric water content corresponding to 25 blows was recorded as the LL. The plastic limit was determined using the 3 mm soil thread method^21^. When the soil thread crack as it reaches the diameter of 3 mm, the soil moisture content is the plastic limit. Gravimetric water contents at the liquid and plastic limits were measured by oven-drying the soil at 105 °C for 48 h. The difference in water content at LL and PL was defined as PI. SOM was determined by the standard Walkley–Black method^20^. Total porosity was calculated from measured ρ_b_, measured ρ_s_ (TP_meas_), and the assumed ρ_s_ of 2.65 Mg m^–3^ (TP_calc_).

### Statistical analysis

One-way ANOVA was used to test the differences in SOM, ρ_s_, ρ_b_, TP, LL, PL and PI under the three different land uses and for each analyzed soil depth. The data was assessed assuming a randomized experiment with three soil profiles from each forest, meadow and arable plot, which represented pseudoreplicates. Many previous studies have used this approach as well [e.g.^6,22^, and references therein]. The assumption is that land use is mostly responsible for the differences in soil properties between the locations because these locations are adjacent to each other and are within the same soil map unit. A simple correlation and regression analysis was undertaken to quantify the relationship among the measured soil properties. SPSS statistical software (IBM SPSS 20 statistical package) was used. The differences in the settings between the treatments were compared by Fisher’s least significant difference (LSD) test at a 5% significance level.

## Results

### Soil organic mater

**Figure 1:**
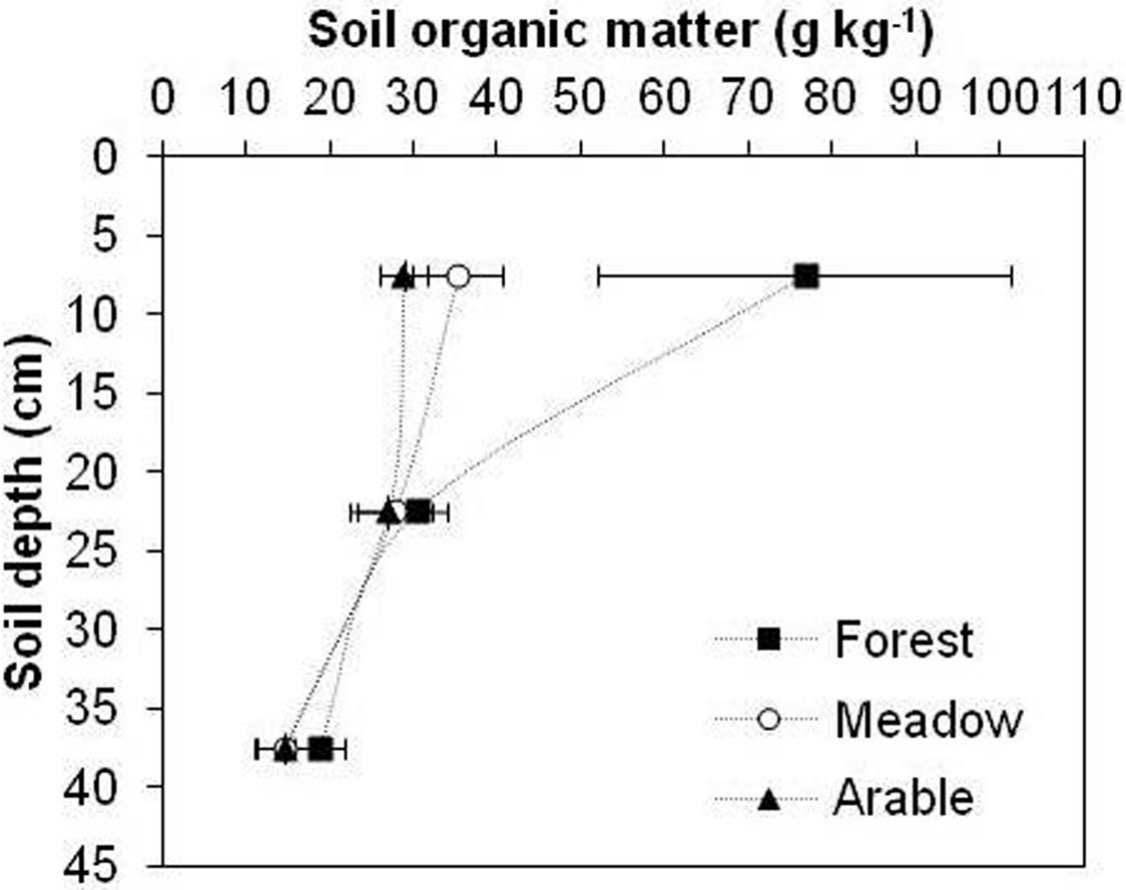
Effect of long-term land use on average SOM at different soil depths. Error bars represent standard deviation of the mean.

The different land use systems had a significant effect on SOM, ρ_s_, ρ_b_, TP and plastic limits of the silty clay soil. The highest average value (~ 77 g kg^–1^) of SOM was observed in forest and the lowest (~ 29 g kg^–1^) in arable soil at the top 15 cm (Fig. 1). The average SOM for the meadow was ~ 35 g kg^–1^ at the same depth. The data presented suggest that the soil organic matter losses after deforestation at 0–45 cm depth decreased for 26% under meadow and for 32% under arable soil, compared with forest.

### Soil density

**Figure 2:**
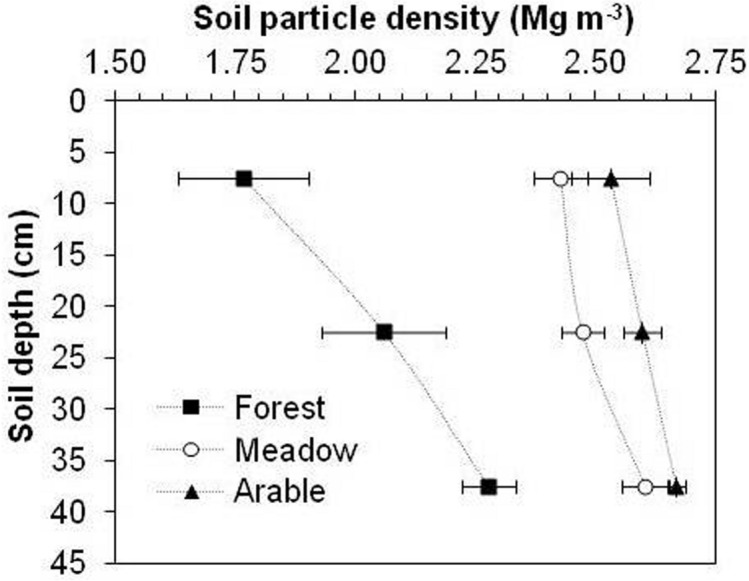
Soil particle density variation as a function of soil depth after different multiyear land uses. Error bars represent standard deviation of the mean.

The native forest soils exhibited a lower average ρ_s_ than the meadow and arable soils regardless of depth (Fig. 2). Forest ρ_s_ was ~ 27% lower at 0–15 cm depth, 17% lower at 15–30 cm and 13% lower at 30–45 cm compared to the meadow, and 30%, 21% and 15% lower than of arable soil, respectively. The meadow ρ_s_ was lower by ~ 4%, 5% and 3%, respectively, that that of arable soil. As expected, ρ_s_ increased with depth in all three treatments and the magnitude of the difference between the meadow and arable soils at a depth of 35–45 cm was relatively large, but not statistically significant.

**Figure 3:**
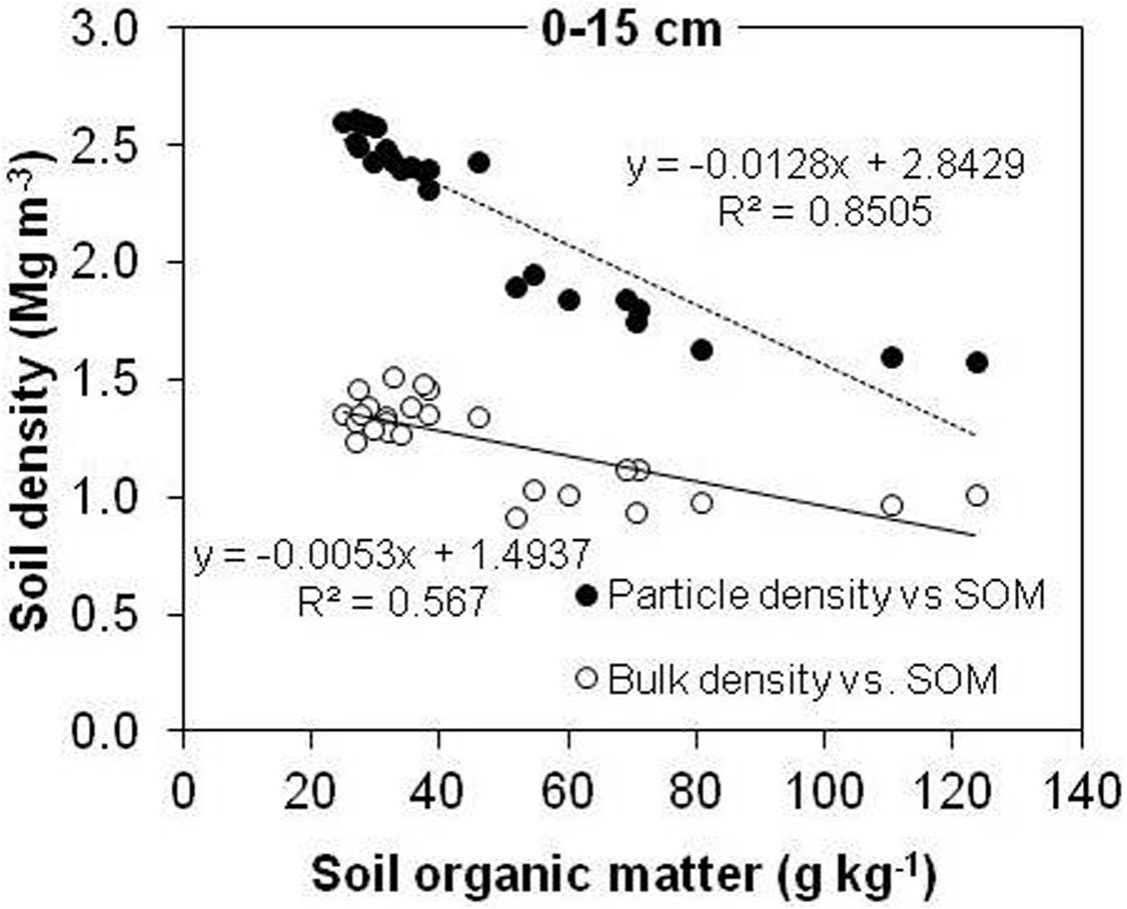
Bulk density and particle density of soil versus SOM at a depth of 0–15 cm under long-term arable land, meadow and forest management.

A statistically significant negative relationship was visible between SOM and ρ_s_in the 0–15 cm depth (Fig. 3)***.***

**Figure 4:**
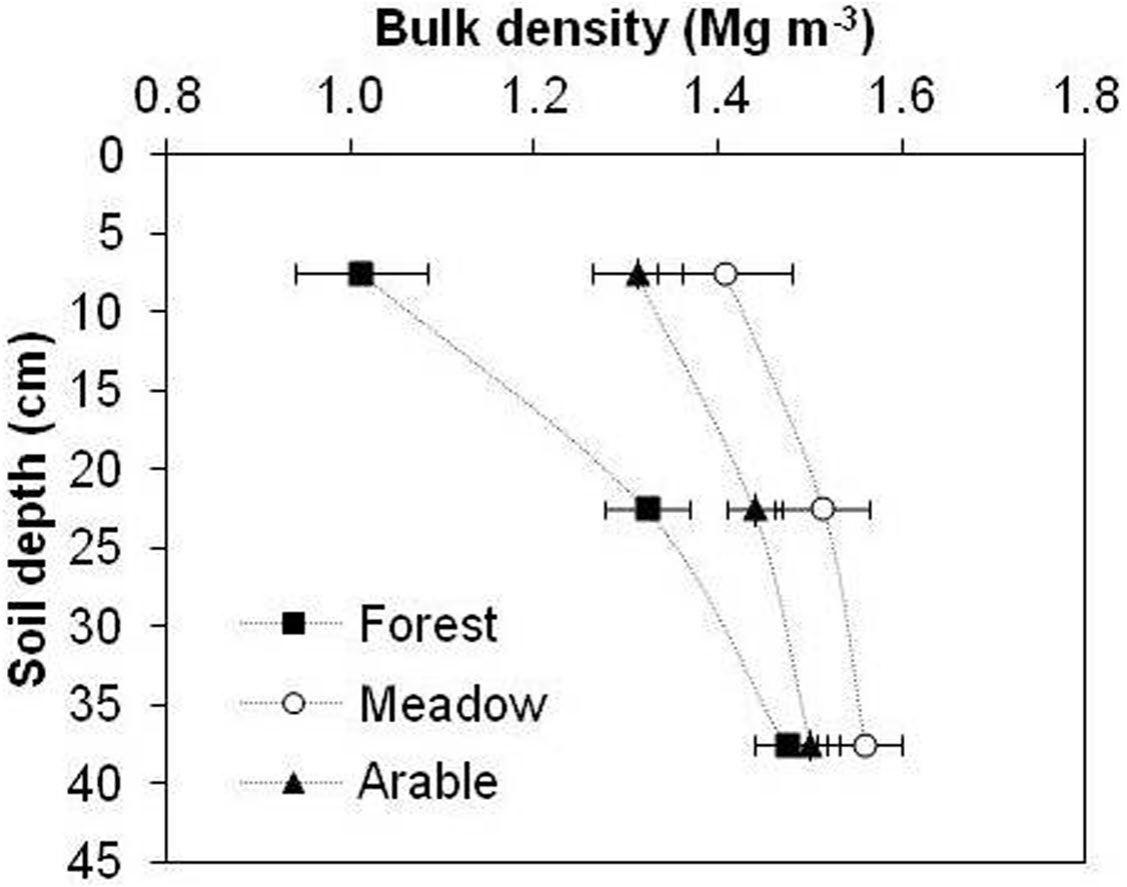
Variation in bulk density as a function of depth under long-term arable land, meadow and forest management. Error bars represent standard deviation of the mean.

The results of the present study show that land use also had a considerable effect on ρ_b_ (Fig. 4), which was used as an important input for TP estimation. It was much higher in the case of meadow than forest or arable soil, at all three depths. It ranged from 1.01–1.48 g cm^–3^ in forest, 1.41–1.56 g cm^–3^ in meadow and 1.31–1.50 g cm^–3^ in arable soil at 0–45 cm depth. A statistically significant negative relationship was visible between SOM and ρ_b_ in the 0–15 cm depth (Fig. 3).

### Soil porosity

**Figure 5:**
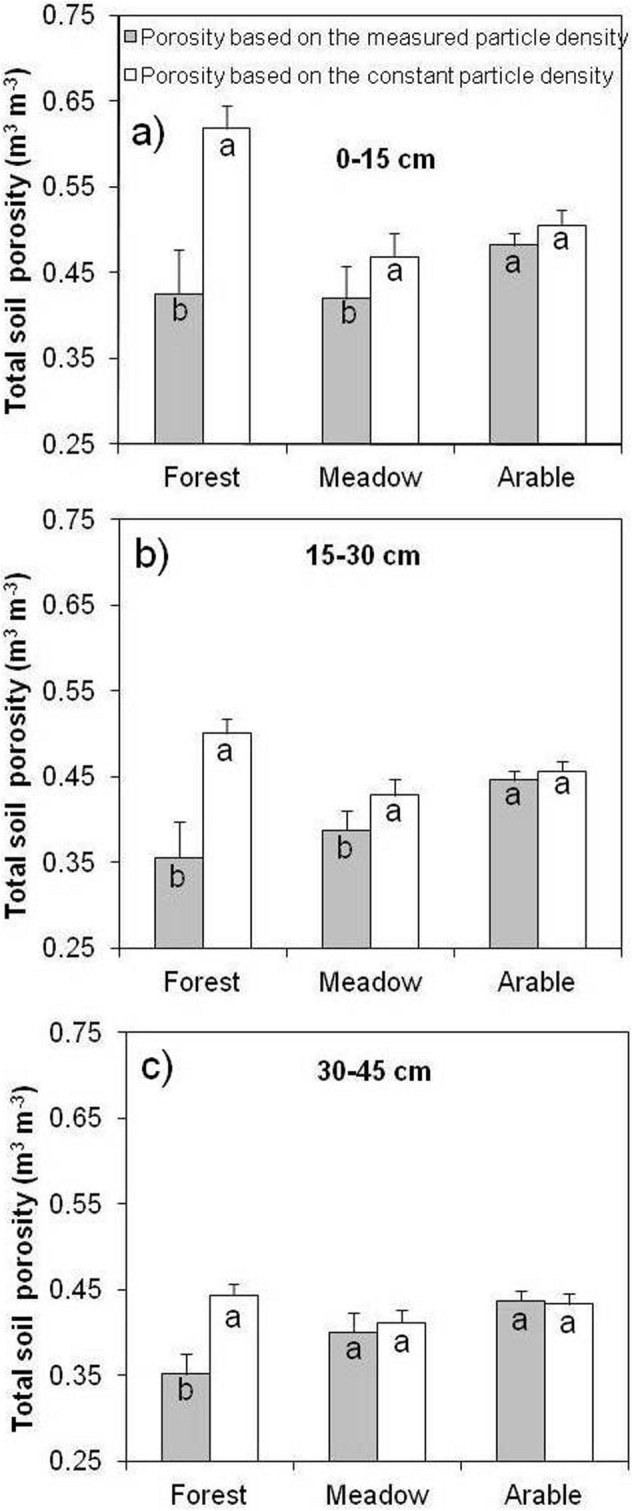
Total soil porosity determined using assumed (2.65 Mg m^–3^) versus measured soil particle density under different long-term land uses at three depth intervals: (**a**) 0–15 cm, (**b**) 15–30 cm, and (**c**) 30–45 cm. Different letters in the same treatment stand for statistically significant differences between two porosities at a probability level of 0.05. Error bars represent standard deviation of the mean.

The TP_meas_ of forest soils differed considerably from TP_calc_ at all three depths (Fig. 5). In contrast, no such differences were found for arable soils at the three depths (0–10, 10–20 and 20–30 cm). In the case of the meadows, there was a statistically significant difference down to a depth of 30 cm. The average TP_calc_ was always higher than TP_meas_, regardless of management practice, except for plowland at 30–45 cm depth (Fig. 5c). Forest soils exhibited the largest discrepancies between TP_calc_ and TP_meas_, and arable soils the lowest. Forest TP_meas_ was 31% lower than TP_calc_ at 0–15 cm depth, while meadow and arable soil TP_meas_ were 10% and 4% lower in the topsoil, respectively (Fig. 5a). The ratio of TP_meas_ to TP_calc_ under all three land uses generally decreased with depth, which was associated with changes in ρ_b_ and ρ_s_, caused by the different land uses.

**Figure 6:**
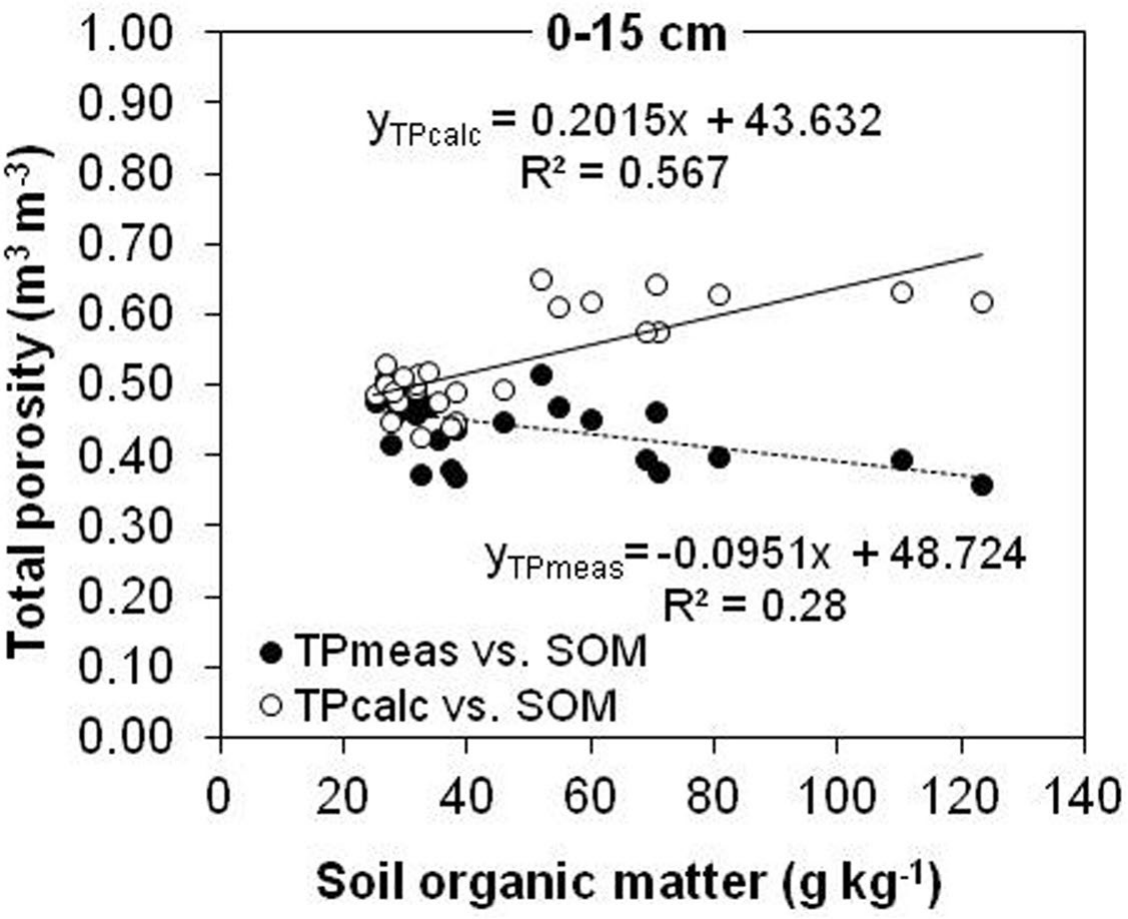
Relationships of measured (TP_meas_) and calculated (TP_calc_) total porosity with SOM content for the 0–15 cm depth.

A statistically significant positive relationship was visible between TP_calc_ and SOM in the 0–15 cm depth (Fig. 6). In contrast, TP_meas_ was the negative and weaker related with SOM content.

### Plastic limits

**Figure 7:**
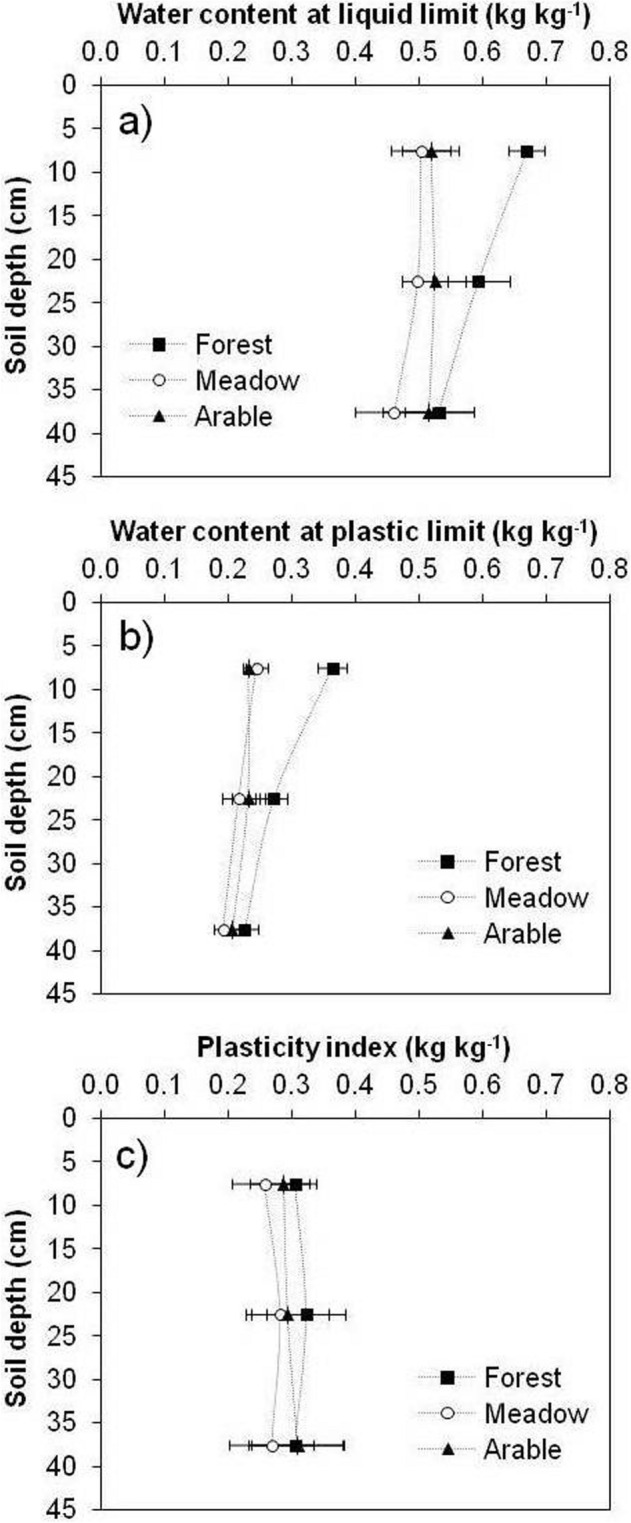
Water content at liquid limit (**a**), plastic limit (**b**) and plasticity index (**c**), at three depths of forest, meadow and arable soils. Error bars represent standard deviation of the mean.

Land use had a statistically significant effect on plastic limits, especially in the surface soil (0–30 cm) (Fig. 7). The differences in water content of the forest soils at LL and PL were significantly higher than in the case of meadow and arable soils at 0–30 cm (Fig. 7a,b). At LL, forest soil retained 29% and 16% more water than the meadow, and 33% and 13% more than the arable soil, at 0–15 cm and 15–30 cm, respectively. LL at 30–45 cm depth significantly differed between the meadow and arable soil (15%), and the arable soil and forest (12%).

The water content of the forest soil at PL was 49% and 25% higher than in the meadow, and 57% and 17% higher than in arable soil, at 0–15 (Fig. 7b) and 15–30 cm depths, respectively. In subsoil (30–45 cm), there was a statistically significant difference in PL between forest and meadow (17%), and forest and arable soil (9%).

In the present study, land use had significant effect on PI at 0–45 cm. On average, PI of the forest was 19% and 7% higher than of the meadow and arable soils, respectively, at the top 15 cm of soil (Fig. 7c). At 15–30 cm depth, the forest PI was 15% higher than that of the meadow and 10% higher than of the arable soil. The arable soil PI was 11% and 4% higher than of the meadow at 0–15 cm and 15–30 cm depths, respectively. On average, the meadow soil PI was ~ 15% lower than of the forest and arable soils at 30–45 cm.

**Figure 8:**
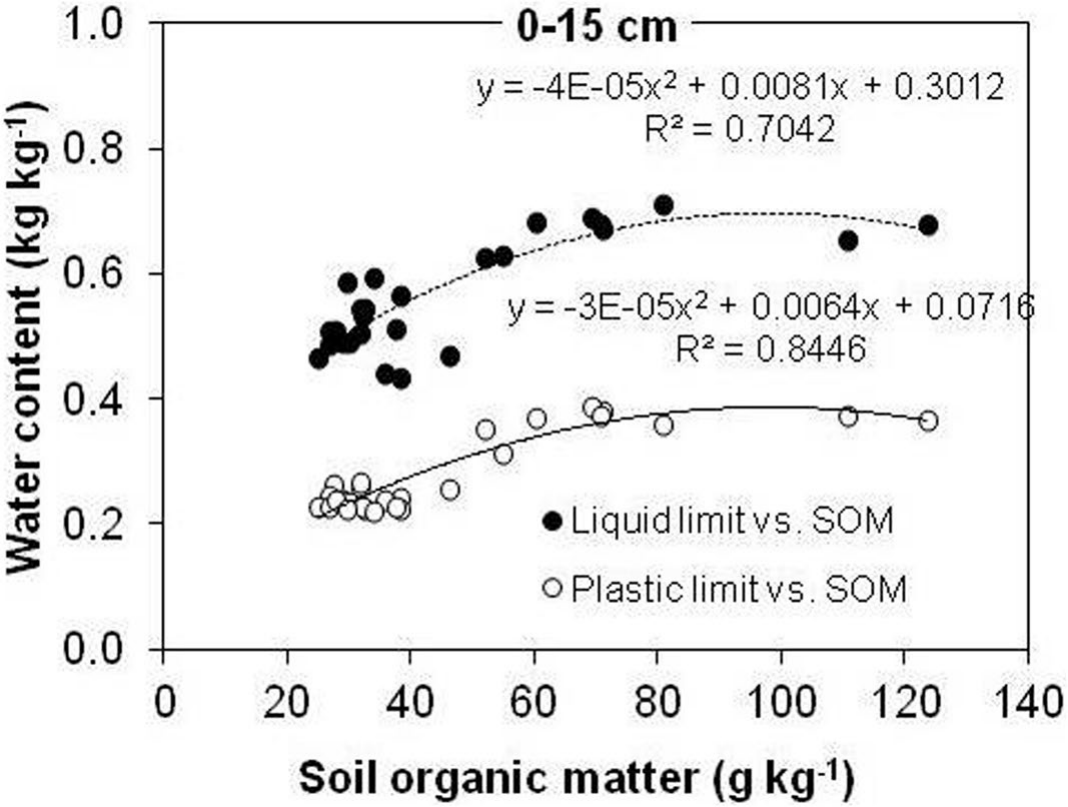
Effect of soil organic matter on liquid limit and plastic limit at a soil depth of 0–15 cm for three different land uses. The *R*^2^ were significant at the 0.05 probability level.

Effect of SOM content on LL and PL is presented in Fig. 8.

### SOM content versus physical and rheological soil properties

SOM plays a very important role in the determined soil properties. In the present study, SOM content were significantly (*p* < 0.01) correlated (Table 1) with ρ_s_ (*r* = − 0.92), ρ_b_ (*r* = − 0.75), LL (*r* = 0.75), PL (*r* = 0.84), TP_meas_ (*r* = − 0.53) and TP_calc_ (*r* = 0.75) at 0–15 cm depth. PI was not correlated with SOM content. There was moderate positive correlation between ρ_s_ and TP_meas_ (*r* = 0.44, *p* < 0.05) and very strong a negative correlation between ρ_s_ and TP_calc_ (*r* = − 0.86, *p* < 0.01). TP_meas_ was not correlated with ρ_b_. The correlations between SOM content and the determined soil properties were weaker in the case of 15–30 cm and 30–45 cm horizons than at 0–15 cm (data not shown), which is probably due to smaller differences in SOM between the different land uses. A perfect negative correlation was found between ρ_b_ and TP_calc_. Both LL and PL better correlated with clay (*r* = 0.84, *r* = 0.79, respectively, *p* < 0.01) then with SOM content (Table 1). PL showed a weaker correlation with clay (*r* = 0.55, *p* < 0.01) than with SOM content (*r* = 0.84, *p* < 0.01).

**Table 1:** Correlation coefficients for relationships between the studied parameters at a depth of 0–15 cm (*n* = 27).

	SOM (g kg^–1^)	ρ_s_ (Mg m^–3^)	ρ_b_ (Mg m^–3^)	LL (kg kg^–1^)	PL (kg kg^–1^)	PI (kg kg^–1^)	TP_meas_ (m^3^ m^–3^)	TP_calc_ (m^3^ m^–3^)	Clay (g kg^–1^)
SOM (g kg^–1^)	1	− .92**	− .75**	.75**	.84**	.26 ns	− .53**	.75**	.56**
ρ_s_ (Mg m^–3^)		1	.86**	− .89**	− .93**	− .38 ns	.44*	− .86**	− .64**
ρ_b_ (Mg m^–3^)			1	− .80**	− .87**	− .31 ns	− .06 ns	− 1.00**	− .58**
LL (kg kg^–1^)				1	.84**	.70**	− .33 ns	.81**	.84**
PL (kg kg^–1^)					1	.20 ns	− .31 ns	.87**	.55**
PI (kg kg^–1^)						1	− .18 ns	.31 ns	.79**
TP_meas_ (m^3^ m^–3^)							1	.06	− .25 ns
TP_calc_ (m^3^ m^–3^)								1	.58**
Clay (g kg^–1^)									1

SOM, soil organic matter; ρ_s_, particle density; ρ_b_, bulk density; LL, liquid limit; PL, plastic limit, PI, plasticity index, TP_meas_, measured soil total porosity; TP_calc_, calculated soil total porosity; ns, non significant.

*, **Significant at 95% and 99% probability levels, respectively.

## Discussion

Land-use changes and subsequent changes in SOM have strongly affected soil physical and rheological properties. The conversion of the natural deciduous forests to other land systems resulted in significant reduction in content of SOM only in the surface soil (0–15 cm) (Fig. 1). Statistically significant differences in SOM were established between the forest and meadow and the forest and arable soil. There was no statistically significant difference between the meadow and arable soil. In general, SOM is assumed to be greater in forests than in meadow and arable soils due to larger inputs and less intense decomposition of SOM. The export of crop residues and lack of manure application have caused lower contents of SOM in the arable and meadow soils. Furthermore, low SOM in the arable soil may be due to the improper management practices adopted by farmers, which mainly burn crop residual after harvesting. Another important factor for the lower SOM in arable soil is the tillage pattern because tillage increases aeration^23^. In contrast to the present study, Saha et al.^24^ reported that the grassland in the topsoil layer (0–15 cm), displayed the highest concentration of SOM (13.2 g kg^–1^), followed by forest land (10.2 g kg^–1^) and cultivated (7.23 g kg^–1^) soils. Similar to our study, Smith et al.^25^ and Villarino et al.^26^ showed that the conversion of native vegetation to agricultural systems strongly affected reduction (9–25%) in SOM. The magnitude of the decrease in SOM in their studies was not as large as in our study (26–32%), probably due to differences in soil type, texture, organic matter source, sampling depth, and soil-crop management.

The average ρ_s_ of arable topsoil (0–15 cm) in the present research was lower than the assumed interval of 2.60–2.70 Mg m^–3^. The ρ_s_ range of the forest soil was always lower than assumed, at all three depths. However, in the case of the meadow, it was lower than assumed down to a depth of 30 cm. Similar to the present study, other researchers^1,6,27^ reported lower than assumed values of ρ_s_ for the topsoils they studied.

The lowest ρ_s_ (Fig. 2) and ρ_b_ (Fig. 4) in the forest soil could be attributed to the corresponding high SOM contents (Fig. 1). The results of the present study are consistent with those reported by Blanco-Canqui et al.^6^, where forest soil ρ_s_ is much lower than that of meadow and arable silt loam soil in Coshocton County, OH. Similar to the present research, Sparling et al.^1^ found that ρ_s_ of plowed soils was somewhat higher (2.53 Mg m^–3^) than that of indigenous forest (2.40 Mg m^–3^) and pastures (2.42 Mg m^–3^) on silt loam soil in New Zealand.

In this study the differences in ρ_s_ between the different land uses were considerably larger than those reported by Sparling et al.^1^, probably because of the duration of soil management and SOM concentration. They compared differences in ρ_s_ for soils after a maximum of 50 years of management, whereas the present study addresses continuous management for > 100 years. Multiyear (> 50 year) studies that compare ρ_s_ between different land uses are relatively rare. In the present study, a much lower ρ_s_ of forest soils than of meadow or tilled soils was expected because of large amounts of organic matter in forest floors. Information in the literature about the effect of forest management on ρ_s_ varies to a large extent. For example, the results of the present study are similar to those of Blanco-Canqui et al.^6^, who reported similar values of ρ_s_ (1.79–2.40 Mg m^–3^) at a depth of 0–30 cm of silt loam forest soils with 2.6–10.8% organic matter content. In contrast to our results, Saprling et al.^1^ determined much higher average values of ρ_s_ (ρ_s_ = 2.40 Mg m^–3^, SOM content = 12.46%) in topsoils (0–7.5 cm) under long-term (> 50 years) native forests in New Zealand. Their results indicate that the average ρ_s_ of forest soils was statistically much lower than of tilled soils (ρ_s_ = 2.53 Mg m^–3^, SOM content = 9.76%), but there was no statistically significant difference between forest and pasture (ρ_s_ = 2.42 Mg m^–3^) soils.

These inconsistent values of ρ_s_ of forest soils might be attributable to differences in SOM content, forest type, soil type, texture, sampling depth, climate conditions, and measurement methods. Furthermore, dissimilar mineralogy could be another factor. The results of our study show that changes in SOM content due to different land uses can indeed have large and significant effects on ρ_s_ as hypothesized.

In the present research, the considerable differences in SOM explained the large difference in ρ_s_ between the land uses. In fact, the linear regression in Fig. 3 indicates that SOM variation explains ~ 85% of the ρ_s_ variation at 0–15 cm soil depth. Figure 3 shows that the effect of SOM content on ρ_s_ is much larger than the effect on ρ_b_ (~ 57%). Our results suggested that ρ_s_ is more controlled by SOM content while ρ_b_ seems to be affected by SOM and also with other factors. Hillel^8^ attributed the decrease in ρ_s_ with increasing SOM to the dilution effect of mineral soil particles. Moreover, SOM usually presents lower particle density than mineral fraction. In the present study, the negative linear relationship between ρ_s_ and SOM (*R*^2^ = 0.85, *n* = 27) at 0–15 cm (Fig. 3) was much stronger than that reported by Ball et al.^28^ in similar research. Similar to this study, Blanco-Canqui et al.^6^ reported a negative linear relationship between ρ_s_ and SOC (*R*^2^ = 0.75–0.87, *n* = 30) in the case of silt loam soil at 0–10 cm. Also, Li et al.^29^ reveal a negative relationship (*R*^2^ = 0.76, *p* < 0 0.01, *n* = 27) between ρ_s_ and SOM content in alpine region of China. These inconsistencies between the various studies might be related to different management durations and practices. The relationships between ρ_s_ and SOM were weaker in the case of 15–30 cm and 30–45 cm soil layers (data not shown), which is probably due to smaller differences in SOM between the different land uses. According to Skopp^30^, the significant and sensitive responses of ρ_s_ to land use changes and SOM content suggested that ρ_s_ could be used as a parameter to reflect the influence of soil management on carbon pool.

The results of the present study indicate that the use of the standard value of ρ_s_ to assess soil porosity overestimates the “real or actual” porosity of the studied soils and could be misleading about the effect of land use on TP. According to Redding et al.^31^, the assumed ρ_s_ value of 2.65 Mg m^–3^ can only be used for soils with a high quartz sand content and low SOM. Our results indicate how important it is to determine ρ_s_ in each routine analysis of physical soil properties and particularly particle size analyses by the sedimentation method, given that ρ_s_ can vary considerably as a function of SOM change.

The loss of SOM by the conversion of the forest into agricultural systems probably caused a statistically significant higher ρ_b_ in meadow and arable soils (Fig. 4). Furthermore, following conversion from the forest, a decline in soil aggregation resulted in the increased ρ_b_ in arable soils. This process could make worse by the continuous use of heavy machinery for soil tillage. The relative high ρ_b_ in the arable soil after 100 years of continuos cropping indicates the occurrence of soil compactness, which directly impels decreased TP. No-tillage in meadow soil may lead to soil compaction due to the lack of soil disturbance and the use of machinery for field operations (e.g., mowing, baling and fertilizer application).

Contrary to our study, Sparling et al.^1^ found no statistically significant differences between ρ_b_ of forest, pasture and arable topsoil in New Zealand. However, Blanco–Canqui et al.^6^ showed that different land uses have a statistically significant impact on ρ_b_, especially at the top 20 cm.

Padalia et al.^32^ obtained strong positive correlation between ρ_b_ and SOM whereas other researchers^6,29,33^ indicated that with SOM increase, ρ_b_ of the soil decrease. In this study, ρ_b_ showed a significant negative correlation (*r* =  − 0.75) with SOM (Table 1). Our results are consistent with other researches^6,29^.

In this study, the total porosity decreased in arable and meadow soils as a coupled result of soil compaction caused by tillage and trampling by traffic, and decline of the SOM content. A greater decrease in ρ_s_ than in ρ_b_ by a unit increase in SOM (Fig. 3) resulted in a significant linear increase in TP_calc_ with increasing total SOM (Fig. 6). Similarly, Li et al.^29^ reported that calculated porosity significantly increased (*r* = 0.71, *p* < 0.01, *n* = 27) with increasing SOM in silt loam soil. In the present study, TP_meas_ decreased linearly with increasing SOM in the surface 15 cm of soils (Fig. 6).

Given that there were no statistically significant differences between the clay content of the forest, meadow and arable soils (data not shown), the differences in LL and PL were likely associated with different SOM content of a relatively high water absorption capacity. Zhang^15^ reported that SOM increases the specific surface area of the soil, leading to increased water retention and thus higher LL and PL. Other studies have also shown that LL and PL increase with increasing SOM^6,16,34^.

Similar to these results, Blanco-Canqui et al.^6^ found no statistically significant difference in PI between silt loam soils under forest and pasture. Contrary to our findings, Zolfaghari et al.^35^ reported a statistically significant difference in PI among land uses in Entisols, Inceptisols and Vertisols of western Iran. Given that plastic limits are sensitive to land use, they could also serve as indicators of physical soil quality, for determining the optimal water content for traffic and tillage.

A quadratic relationship between LL/PL water content and SOM (Fig. 8) was established at a depth of 0–15 cm. SOM variations explained 70% of LL, 84% of PL and only ~ 10% of PI variability (data not shown). A strong relationship between SOM and LL (Fig. 8) indicates that the soils with high SOM content require higher amounts of water to pass from plastic to liquid state and that change in water holding capacity at LL and PL were closely linked to changes in SOM.

In the subsurface soil layers (15–30 and 30–45 cm), there was a weaker relationship between SOM and the plastic limits. SOM variations explained 23% and 24% of LL variability and 1% and 16% of PL variability, at 15–30 cm and 30–45 cm, respectively (data not shown).

The quadratic regression curves of LL and PL in Fig. 8 rise rapidly to ~ 90 g kg^–1^ of SOM, and then drop slightly as SOM increases further, which is slightly higher then reported by Blanco-Canqui et al.^6^ (~ 83 g kg^–1^ of SOM). The decrease in the water content at LL and PL above ~ 90 g kg^–1^ of SOM could be a result of qualitative differences in SOM between forest and agricultural soils^6^. According to those researchers, contrary to forest soils, SOM of agricultural soils has a stronger bond with the mineral fraction, which accounts for its effect on soil consistency. As visible from the correlations reported in Table 1, SOM content strongly relates with the LL and PL in this soil.

Hemmat et al.^16^ established a strong, statistically significant and positive linear relationship between SOC and LL/PL in topsoil (0–20 cm) in central Iran, composed of fine-loamy Calcaric Cambisols. Similar results were also reported by Abdi et al.^36^ where the plasticity properties (LL and PL) of the clay soil, except for the PI, increased linearly with an increase in organic matter content. Contrary to those researchers, Keller and Dexter^17^ reported a weak positive correlation between SOM and LL/PL, while there was no significant effect of SOM on PI for agricultural soils covering a wide range of soil types in many countries. Stanchi et al.^37^ found no correlation between LL, PI and the SOM content, while they observed a positive relationship between PL and SOM of clayey, organic C-rich mountain soils in the Ligurian Alps (NW-Italy).

In contrast, Blanco-Canqui et al.^6^ reported a statistically significant correlation between PI and SOM, whereas our results agree with those of Ball et al.^28^, Keller and Dexter^17^ and Abdi et al.^36^. In addition, Lal and Shukla^38^ reported that in most mineral soils with an SOM content of less than 5%, LL and PL increased with increasing SOM. Therefore, in their opinion, SOM need not have an effect on PI. According to De Jong^14^, these discrepancies are attributable to the duration and type of management, soil parent material, clay content and mineralogy, and SOM type and nature.

Data on soil plasticity (Figs. 7, 8) showed that arable and meadow soils become compacted at lower water contents than forest soils. These findings suggest that arable and meadow soils can be trafficked at relatively lower water contents and have higher susceptibility to compaction than forest soils at the same water content. In the topsoils (0–15 cm), LL and PL showed significant positive linear correlation with the SOM content, indicating that the SOM content, despite its modest amounts in agricultural soils, plays a major role in the investigated pedo-climatic conditions regarding the soil consistency conservation.

The negative correlation of ρ_s,_ ρ_b_ and TP_meas_ with SOM content suggests that the decrease in SOM with deforestation was responsible for the increase in ρ_s,_ ρ_b_ and TP_meas_ (Table 1). The correlation analyses showed that SOM content was more strongly (*r* =  − 0.92, *p* < 0.01) correlated with ρ_s_ than with ρ_b_ (*r* =  − 0.75, *p* < 0.01).

## Conclusions

This study suggests that conversion of native deciduous forests to agricultural soils increase the susceptibility of soil to compaction by decreasing SOM content in the top 15 cm of the soil. Bulk density and water content at plastic limits decreased, whereas the particle density increased with a decrease in SOM content in topsoil. Calculated total porosity overestimated the “true” porosity. This necessitated measuring of ρ_s_ for accurate determination of soil properties associated with ρ_s_. Clear linear relationship was visible between SOM content and ρ_s_, ρ_b_, TP_calc_ and TP_meas_, in studied environment. In addition, there was a strong curved relationship between SOM and LL/PL. Water content at plastic and liquid limits does not rise above ~ 90 g kg^–1^ of SOM, whereas PI was unaffected by topsoil SOM. The present research indicated that SOM resulting from the conversion of native deciduous forests into meadows and arable land had a statistically significant effect on soil properties and water content interval in which the soil was plastic and featured optimal conditions for tillage and traffic, as hypothesized in the aims. Degradation of the investigated soil physical properties is closely linked with the reduction of SOM content which is a consequence of deforestation and different management.
